# A Novel Hepadnavirus Identified in an Immunocompromised Domestic Cat in Australia

**DOI:** 10.3390/v10050269

**Published:** 2018-05-17

**Authors:** Mahdis Aghazadeh, Mang Shi, Vanessa R. Barrs, Alicia J. McLuckie, Scott A. Lindsay, Barbara Jameson, Bronte Hampson, Edward C. Holmes, Julia A. Beatty

**Affiliations:** 1 Sydney School of Veterinary Science, Faculty of Science, University of Sydney, Sydney, NSW 2006, Australia; mahdis.aghazadeh@sydney.edu.au (M.A.); vanessa.barrs@sydney.edu.au (V.R.B.); amcl8135@uni.sydney.edu.au (A.J.M.); bham3841@uni.sydney.edu.au (B.H.); 2 School of Life and Environmental Sciences and Sydney Medical School, Charles Perkins Centre, University of Sydney, Sydney, NSW 2006, Australia; mang.shi@sydney.edu.au (M.S.); Edward.Holmes@sydney.edu.au (E.C.H.); 3 Marie Bashir Institute for Infectious Diseases and Biosecurity, The University of Sydney, Sydney, NSW 2006, Australia; 4 School of Animal and Veterinary Sciences, Faculty of Science, The University of Adelaide, Adelaide, Roseworthy, SA 5371, Australia; scott.lindsay@adelaide.edu.au; 5 Vets@Acacia Gardens, Quakers Hill, NSW 2006, Australia; info@acaciagardensvet.com.au

**Keywords:** virus, hepadnavirus, *Orthohepadnavirus*, immuosuppression, hepatitis B, domestic cat, feline, carnivore, pathogen discovery

## Abstract

High-throughput transcriptome sequencing allows for the unbiased detection of viruses in host tissues. The application of this technique to immunosuppressed animals facilitates the detection of viruses that might otherwise be excluded or contained in immunocompetent individuals. To identify potential viral pathogens infecting domestic cats we performed high-throughput transcriptome sequencing of tissues from cats infected with feline immunodeficiency virus (FIV). A novel member of the *Hepadnaviridae*, tentatively named domestic cat hepadnavirus, was discovered in a lymphoma sample and its complete 3187 bp genome characterized. Phylogenetic analysis placed the domestic cat hepadnavirus as a divergent member of mammalian orthohepadnaviruses that exhibits no close relationship to any other virus. DNA extracted from whole blood from pet cats was positive for the novel hepadnavirus by PCR in 6 of 60 (10%) FIV-infected cats and 2 of 63 (3.2%) FIV-uninfected cats. The higher prevalence of hepadnavirus viraemia detected in FIV-infected cats mirrors that seen in human immunodeficiency virus-infected humans coinfected with hepatitis B virus. In summary, we report the first hepadnavirus infection in a carnivore and the first in a companion animal. The natural history, epidemiology and pathogenic potential of domestic cat hepadnavirus merits additional investigation.

Viruses of the genus *Orthohepadnavirus*, family *Hepadnaviridae*, are partially double-stranded DNA viruses that infect a variety of mammals including primates, bats and rodents. The type species, hepatitis B virus (HBV), is a major public health problem; chronic HBV infection increases the risk of liver diseases including cirrhosis and hepatocellular carcinoma, and an estimated 257 million people are currently living with HBV [1].

We identified a new hepadnavirus in a domestic cat from Australia. In 2016, a seven year-old male-neutered domestic shorthair cat was presented for vomiting and weight loss. On physical examination a large mid-abdominal mass was palpated. Fine needle aspirate cytology of the mass was consistent with large cell lymphoma. The owners requested euthanasia and necropsy and consented to tissue collection approved by the University of Sydney animal ethics committee, (2014/626). Multicentric, large cell, high-grade, B-cell lymphoma was diagnosed on immunohistopathology of formalin-fixed paraffin embedded tissue. Infection with the immunosuppressive lentivirus, feline immunodeficiency virus (FIV), was diagnosed by serology for anti-FIV antibody (Witness FIV/FeLV, Zoetis) and PCR of tumour DNA confirmed by sequencing as described previously [2]. Lymphoma tissue from this case was utilized in a virus discovery project.

Total RNA extracted from frozen lymphoma was enriched for non-ribosomal RNA and Illumina TruSeq paired-end libraries were prepared. Briefly, 30 mg of lymphoma was homogenised using 1.4 mm ceramic beads (OMNI Homogeniser, Kennesaw, GA, USA), total RNA was extracted using the Qiagen RNeasy mini kit (Qiagen, Hilden, Germany) followed by on-column DNA removal to remove genomic DNA. RNA quality, examined using an Agilent Bioanalyzer 2100 (Agilent Technologies, Mulgrave, VIC, Australia), demonstrated RNA integrity numbers greater than eight. Library preparation was performed using a TruSeq RNA library preparation kit (Illumina, San Diego, CA, USA) and ribosomal RNA was removed using a Ribo-Zero gold epidemiology kit (Illumina, USA). RNA sequencing of 100 bp pair-end libraries on an Illumina HiSeq 2500 platform (San Diego, CA, USA) yielded 321,297,408 total reads. Sequencing reads were uploaded onto the NCBI Sequence Read Archive (SRA) database under the BioProject submission SUB3718346. 

To identify potential viral transcripts, sequencing reads were assembled de novo using the Trinity software (version 2.1.1) [3]. The resulting contigs were then blasted against the non-redundant nucleotide database (nt) using blastn program with an E-value threshold of 1 × 10^−10^ and against the non-redundant protein database (nr) using Diamond version 0.8.28.90 with an E-value threshold of 1 × 10^−4^ [4]. To discover viruses at low abundance, we performed Diamond blastx analyses on sequence reads against the nr database. The virus-associated contigs and reads were recovered based on the taxonomic annotation of each blast hit. Each positive hit was subsequently confirmed by manually inspecting the nucleotide or amino acid sequence alignment with their closest relatives. To exclude the potential presence of endogenous virus (EVEs) in the blast results, we also blasted the reads/contigs against the cat genome sequences available at the reference genome database and the whole genome shotgun database. In total, eight HBV-like reads were identified that shared 73–94% amino acid identity with the core protein, surface protein, and polymerase of known hepadnaviruses. This suggested the presence of a novel virus that was divergent from currently known hepadnaviruses. 

To confirm the presence of the hepadnavirus, DNA was extracted from frozen lymphoma and examined with a panhepadnavirus PCR assay targeting the polymerase ORF [5]. A 250 bp product was obtained and sequenced, thereby validating the RNA sequencing. To amplify the entire genome of the novel hepadnavirus from lymphoma tissue, a five primer-set PCR was designed based on recovered reads (Table 1). PCR reactions were performed using Bioline MyTaq HotStart polymerase (Bioline, Australia) based on the manufacturer’s recommended protocol, using 200–500 ng of template DNA, 1× MyTaq buffer, a final primer concentration of 0.4 µM in a 50 µL reaction and an annealing temperature ranging from 55 to 58 °C. For primer sets Hgap-F/R and Cir5-F/Cir4-R a final primer concentration of 0.3 µM was used. The amplicons were sequenced (Macrogen, Seoul, South Korea) and analysed using Geneious version 11.2.0 and the entire 3187 bp circular genome was assembled (Figure 1). The genome contained the polymerase (P), surface (S), core (C) and X ORFs typical of orthohepadnaviruses. Notably, the complete P protein exhibited between 63.2% to 68.7% amino acid similarity to known orthohepadnaviruses. Such a large genetic distance merits assignment of a new species within the genus *Orthohepadnavirus*, which we tentatively named domestic cat hepadnavirus. The complete genome sequence of domestic cat hepadnavirus has been deposited in GenBank, accession number MH307930.

To determine the evolutionary relationships of domestic cat hepadnavirus, we compared it to reference sequences of P proteins from the entire *Hepadnaviridae* family downloaded from GenBank. To show the phylogenetic diversity of vertebrate hepadnavirusues in the P protein tree, homologous sequences representative of the viral diversity in other vertebrates [6,7] were added to the alignment. In the case of the more divergent C and S ORFs, data sets of orthohepadnaviruses only were constructed, utilizing the hepadnavirus from the bluegill (GenBank accession KX058433) as an outgroup [6]. However, because of the small size of the X ORF gene alignment, the lack of an appropriate outgroup for this ORF, and because it is largely contained within the other ORFs, the X ORF was excluded from the analysis. All amino acid sequences were aligned using MAFFT (version 7) [8], and all ambiguously aligned regions were removed using TrimAl [9]. This process resulted in final data sets of the following sizes: P protein = 30 taxa, 419 amino acids; C protein = 16 taxa, 182 amino acids; S ORF = 16 taxa, 255 amino acids.

Phylogenetic trees of all three data sets were inferred using the maximum likelihood approach available in PhyML 3.0, employing the LG + Γ model of amino acid substitution and a SPR branch-swapping algorithm [6]. In the case of the P ORF phylogeny (Figure 2A) it is striking that although domestic cat hepadnavirus clearly groups with the mammalian orthohepadnaviruses, it was the most basal member of this group, including those viruses recently identified in bats [10], and exhibited no close phylogenetic relationship to any other hepadnavirus [11]. This divergent position was also well supported by SH-like branch supports (value of 0.84) separating domestic cat hepadnavirus from the other mammalian hepadnaviruses. Less phylogenetic resolution (i.e., lower SH-like branch supports at key nodes) and differing topologies (such as the position of the tent-making bat) are apparent in the shorter C (Figure 2B) and S (Figure 2C) ORF alignments, although domestic cat hepadnavirus was consistently distinct from all other orthohepadnaviruses. Although this analysis clearly shows that domestic cat hepadnavirus is evolutionarily distinct, its exact phylogenetic position is uncertain.

Hepadnaviruses are typically hepatotropic, although viraemia is common in immunosuppressed hosts [12]. To investigate whether this immunosuppressed cat was viraemic for the novel hepadnavirus, PCR was performed on DNA extracted from autologous frozen whole blood using primer set Hgap-F/R (Table 1). A positive result on PCR was confirmed by sequencing. Next, a molecular survey for hepadnavirus viraemia was performed using DNA extracted from stored whole blood from adult pet cats in Australia (University of Sydney Animal Ethics Committee approvals N00/7-2013/3/6029 and 2016/1002). The novel hepadnavirus was detected in whole blood from 6 of 60 (10%) FIV-infected cats and 2 of 63 (3.2%) uninfected cats. Confirmation of this finding in larger studies is awaited. Nonetheless, it is noteworthy that a similar pattern is seen among HIV infected humans where HBV is a common co-pathogen [13]. For example, in USA and Europe the prevalence of persistent HBV antigenaemia is 1% in the general population compared with 7–10% among HIV-infected homosexual men who are more likely to be exposed to and less likely to clear HBV [14].

In conclusion, we report the first hepadnavirus infection in a member of the mammalian order Carnivora and the first report of a hepadnavirus in a companion species, with hepadnavirus viraemia commonly detected among FIV-infected cats. Future investigation of the natural history and epidemiology of domestic cat hepadnavirus and its impact on feline health is clearly warranted.

## Figures and Tables

**Figure 1:**
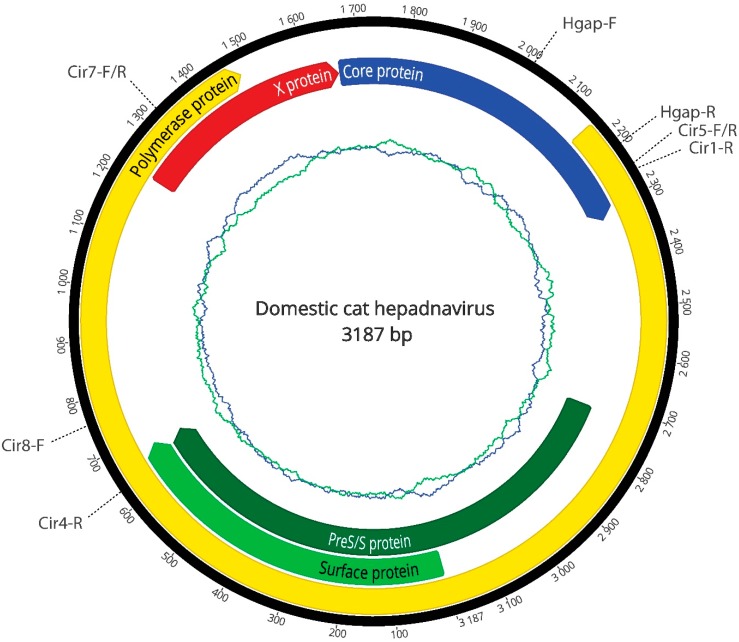
Genome organization of the domestic cat hepadnavirus. The complete genome consists of 3187 bp. The innermost circles represent the GC (blue) and AT (green) content of the genome. The proteins encoded by the polymerase, surface, core and X ORFs are labelled, as are the positions of primers used in this study (Table 1).

**Figure 2:**
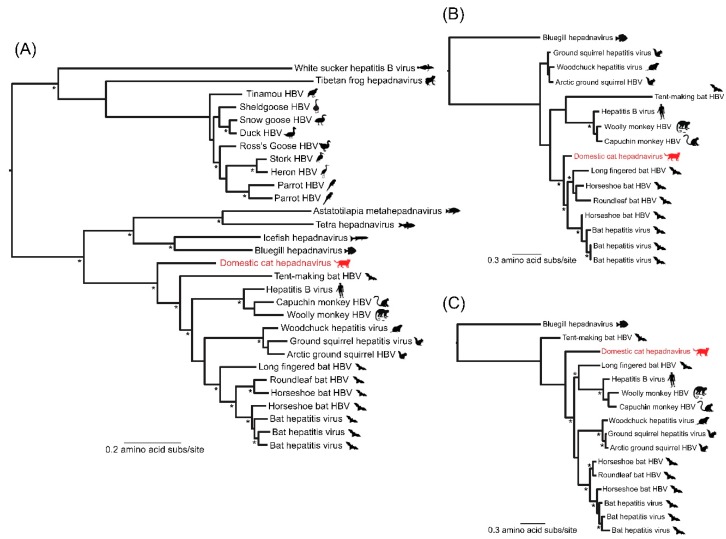
The Phylogenetic position of domestic cat hepadnavirus within the *Hepadnaviridae*. (**A**) Maximum likelihood phylogeny based on the P (polymerase) ORF and incorporating a wide range of vertebrate hepadnaviruses, including Roundleaf bat HBV (YP_009045991), Horseshoe bat HBV (YP_009045995), Long-fingered bat HBV (YP_007677999), Tent-making bat HBV (YP_009045999), Bat HBVs (AVW79974, ARM20233, ARM20221, and AQT40960), Ground squirrel hepatitis virus (NP_040994), Arctic ground squirrel HBV (AAB08032), Woodchuck hepatitis virus (NP_671813); Hepatitis B virus (YP_009173866), Woolly monkey HBV (YP_009175034), Capuchin monkey HBV (AVV68831), Bluegill hepadnavirus (YP_009259541), Astatotilapia metahepadnavirus (ref7), Tetra hepadnavirus (ref7), Icefish hepadnavirus (ref7), Heron HBV (NP_040998), Parrot HBVs (YP_004956864, AFY97702), Ross's goose HBV (YP_024968), Duck HBV (NP_039821), Snow goose HBV (YP_031695), Stork HBV (CAC80811), Sheldgoose HBV (YP_024974), Tinamou HBV (YP_009389524), Tibetan frog hepadnavirus (YP_009259545), White sucker HBV (YP_009165599). This tree was mid-point rooted for clarity only. Phylogenies based on the (**B**) C ORF (**C**) and S ORF of mammalian orthohepadnaviruses and rooted by the homologous sequences found in the bluegill. In all phylogenies the host groups of each virus species are indicated by symbols to the right of the tree and branch lengths are scaled according to the number of amino acid substitutions per site. SH-like support values >0.80 for nodal support are shown by asterisks in all cases.

**Table 1:** Oligonucleotides used to amplify the whole genome of domestic cat hepadnavirus.

Oligonucleotides	Sequence	Size (bp)	Tm (°C)
Cir5-F	5′-TTGGCACCTGGATTCGCA-3′	1400	57
Cir4-R	5′-AGATGTTCCACACTCTTAGCC-3′		
Cir8-F	5′-TTGGCACCTGGATTCGCA-3′	900	58
Cir7-R	5′-CGTAGACGAAGGACACGTC-3′		
Cir7-F	5′-CCATCGATTTACACACTTCCCA-3′	950	57
Cir5-R	5′-TGCGAATCCAGGTGCCAA-3′		
Cir1-R	5′-ATAACCGTATGCTCCGGAAG-3′	1000	55
Hgap-F	5′-GTGCTCTGATAACCGTATGCTC-3′	230	55
Hgap-R	5′-CTAGAATGGCTACATGGGGTTAG-3′		
